# Association of SARS-CoV-2 status and antibiotic-resistant bacteria with inadequate empiric therapy in hospitalized patients: a US multicenter cohort evaluation (July 2019 - October 2021)

**DOI:** 10.1186/s12879-023-08453-z

**Published:** 2023-07-24

**Authors:** Karri A. Bauer, Laura A. Puzniak, Kalvin C. Yu, Kenneth P. Klinker, Janet A. Watts, Pamela A. Moise, Lyn Finelli, Vikas Gupta

**Affiliations:** 1 Merck & Co., Inc, Kenilworth, NJ USA; 2 Becton, Dickinson and Company, 1 Becton Drive, Franklin Lakes, NJ USA

**Keywords:** Antibiotic resistance, SARS-CoV-2, COVID-19, Inadequate empiric therapy, Bacteria

## Abstract

**Background:**

Antibiotic usage and antibiotic resistance (ABR) patterns changed during the COVID-19 pandemic. Inadequate empiric antibiotic therapy (IET) is a significant public health problem and contributes to ABR. We evaluated factors associated with IET before and during the COVID-19 pandemic to determine the impact of the pandemic on antibiotic management.

**Methods:**

This multicenter, retrospective cohort analysis included hospitalized US adults who had a positive bacterial culture (specified gram-positive or gram-negative bacteria) from July 2019 to October 2021 in the BD Insights Research Database. IET was defined as antibacterial therapy within 48 h that was not active against the bacteria. ABR results were based on susceptibility testing and reports from local facilities. Multivariate analysis was used to identify risk factors associated with IET in patients with any positive bacterial culture and ABR-positive cultures, including multidrug-resistant (MDR) bacteria.

**Results:**

Of 278,344 eligible patients in 269 hospitals, 56,733 (20.4%) received IET; rates were higher in patients with ABR-positive (n = 93,252) or MDR-positive (n = 39,000) cultures (34.9% and 45.0%, respectively). Severe acute respiratory syndrome-coronavirus-2 (SARS-CoV-2)-positive patients had significantly higher rates of IET (25.9%) compared with SARS-CoV-2-negative (20.3%) or not tested (19.7%) patients overall and in the ABR and MDR subgroups. Patients with ABR- or MDR-positive cultures had more days of therapy and longer lengths of stay. In multivariate analyses, ABR, MDR, SARS-CoV-2-positive status, respiratory source, and prior admissions were identified as key IET risk factors.

**Conclusions:**

IET remained a persistent problem during the COVID-19 pandemic and occurred at higher rates in patients with ABR/MDR bacteria or a co-SARS-CoV-2 infection.

**Supplementary Information:**

The online version contains supplementary material available at 10.1186/s12879-023-08453-z.

## Background

Patients with signs and symptoms of bacterial infections are typically treated with empiric antibiotic therapy prior to the availability of information on the specific pathogen and antimicrobial susceptibility. Choosing appropriate empiric therapy thus poses a clinical challenge that can have important consequences. Inadequate empiric therapy (IET), defined as antibacterial therapy not active against the identified bacterial pathogen, is associated with increased mortality, hospitalization, and readmission rates, longer length of stay (LOS), additional antibiotic prescriptions, and higher costs [1–7]. Antibiotic resistance (ABR), particularly multi-drug resistance (MDR), is a key driver of IET [6, 7], in part because ABR bacteria have fewer available therapeutic options to utilize prior to diagnostic results.

Among hospitalized patients, approximately 8–20% of patients who test positive for severe acute respiratory syndrome-coronavirus-2 (SARS-CoV-2) have positive cultures for a bacterial co-pathogen, yet 68–76% are treated with antibiotics [8–11]. These high rates of antibiotic use during the COVID-19 pandemic have been observed in hospitalized patients with influenza as well [12]. There are likely multiple reasons for this pattern, including the desire to prevent bacterial pneumonia and secondary infections, reduced diagnostic and antibiotic susceptibility testing in over-taxed healthcare systems, and uncertainty on how best to manage complex patients with a potentially severe respiratory disease [8, 9, 13]. Increased antibiotic use contributes to ABR, thus potentially compounding challenges involved in patient care [14–16]. In one recent study, overall ABR rates per 1000 admissions did not increase in the pandemic compared with pre-pandemic period, but ABR rates were higher in hospitalized SARS-CoV-2-positive patients compared with SARS-CoV-2-negative patients or those not tested for SARS-CoV-2. The largest increase in ABR rates was observed in hospital-onset bacterial cultures [17].

The COVID-19 pandemic has influenced antibiotic usage and ABR, which can lead to changes in IET rates and patient outcomes. We conducted a study of hospitalized US patients prior to and during the COVID-19 pandemic (July 2019 to October 2021) to identify patient and hospital factors associated with IET by ABR status and time period.

## Methods

### Study design

We conducted a multicenter, retrospective cohort analysis of all hospitalized adults aged ≥ 18 years from 269 US facilities in the BD Insights Research Database (Becton, Dickinson and Company, Franklin Lakes, NJ), which includes both small and large medical care facilities in rural and urban areas throughout the United States. This electronic surveillance system and clinical research database has been previously described and encompasses pharmacy, laboratory, administrative data, patient demographics, and admission, discharge, and transfer data feeds [1, 8, 18, 19]. The retrospective, de-identified data set was approved and informed consent requirements were waived by the New England Institutional Review Board (Wellesley, MA, USA; IRB No. 120,180,023).

Eligible patients included subjects with 1 to 365 days inpatient stay, a record of discharge or death between July 1, 2019, and October 30, 2021, a positive bacterial culture, and a record of antibiotic therapy. The pre-SARS-CoV-2 period was defined as July 1, 2019 to February 29, 2020. A culture-positive potential bacterial pathogen was defined as a non-contaminant first positive culture for a specified gram-negative or gram-positive bacteria from respiratory, blood, urine, skin/wound, intraabdominal or other source. Microbiology results likely associated with a contaminant or surveillance culture were excluded by a previously described methodology that uses source, time of collection, microorganism type, and number of microorganisms in a culture to flag likely contaminated samples [20].

The major outcome of interest was identification of risk factors contributing to IET, defined as antibiotic therapy prescribed within 48 h from a positive culture collection that did not cover the bacteria or to which the bacteria was subsequently reported as nonsusceptible (NS; laboratory result of intermediate resistance [I] or resistant [R]). For the purposes of this study, the designation of IET was restricted to patients with no adequate antibiotic agent prescribed within 48 h of a positive culture; a patient who received multiple antibiotics with at least one active therapy was NOT categorized as IET. Days of therapy (DOT) included all days of antibiotic therapy (both active or inactive drugs) associated with the index bacteria hospital admission, either before or after the first positive culture.

For the purposes of comparison, patients were categorized into 3 subgroups: (1) patients with any culture positive for a specified gram-negative or gram-positive bacteria; (2) the subset of culture-positive patients with an ABR-positive culture (as defined below); and (3) the subset of ABR-culture positive patients with an MDR-positive culture (as defined below).

### Antibiotic susceptibility assessments

Gram-negative bacteria included in these analyses were Enterobacterales (*Citrobacter freundii, Escherichia coli, Enterobacter cloacae* complex, *Klebsiella pneumoniae, Klebsiella oxytoca, Klebsiella aerogenes, Morganella morganii, Proteus mirabilis, Providencia stuartii, Serratia marcescens*), *Pseudomonas aeruginosa*, *Acinetobacter* complex, and *Stenotrophomonas maltophilia.* Gram-positive bacteria included in the analyses were *Enterococcus* spp., *Staphylococcus aureus*, and *Streptococcus pneumoniae*.

Antibiotic susceptibility analyses are detailed in Supplementary Section S1. Briefly, ABR in gram-negative bacteria was defined as NS to extended-spectrum cephalosporins (ESC), piperacillin-tazobactam, carbapenems, or fluroquinolones (FQ). MDR in gram-negative bacteria was defined as carbapenem NS, extended-spectrum beta-lactamase producing phenotype, pan-beta-lactam NS, or NS to at least 1 drug in 3 of the following 5 classes: ESC; FQ; carbapenems; aminoglycosides; piperacillin, or piperacillin-tazobactam. For gram-positive bacteria, ABR was defined as *Enterococcus* resistant to vancomycin (VRE), *S. aureus* resistant to methicillin (MRSA), and *S. pneumoniae* NS to penicillin, macrolides, FQ, ESC, or tetracyclines. MDR in gram-positive bacteria was defined as VRE or MRSA. Bacteria defined as MDR were also included in the ABR category.

ABR was identified at the admission level as the first positive culture with any of the ABR bacteria described above. ABR was considered to be community-onset if the first positive ABR event culture was collected ≤ 2 days from admission (with day 1 as day of admission) and was defined as hospital-onset if the culture was collected > 2 days from admission. For patients with an ABR culture, previous susceptible cultures and subsequent ABR or non-ABR cultures with the same or a different pathogen were not evaluated. All results from microbiology testing were obtained from analyses performed by local microbiology labs in the cohort of hospitals included in the BD Insights Research Database. A central laboratory was not used for these analyses.

### Statistical analysis

The potential risk factors for IET considered in the study included: ABR to evaluated antibiotics as described above, MDR, COVID-19 time period (pre-SARS-CoV-2, SARS-CoV-2), SARS-CoV-2 testing status (positive, negative, or not tested), pathogen category (gram-positive, gram-negative, both gram-positive and gram-negative), specific bacteria, clinical factors and characteristics associated with the index bacteria hospital admission including the time before and after culture collection (culture source [respiratory, blood, urine, skin/wound, intraabdominal or other]; onset (community or hospital-onset); DOT; LOS; intensive care unit [ICU] admission; ventilated status; and prior 30- or 90-day admission), underlying clinical condition (sepsis/severe sepsis, renal insufficiency/failure, liver dysfunction, myocardial injury, and cytokine dysregulation syndrome) [1, 21], patient demographics (sex and age), facility characteristics (bed size, urban/rural location, teaching status, and geographic region [US census region]).

In the exploratory phase of the analysis, we performed descriptive analyses of IET for three subgroups based on ABR status (all positive cultures, ABR-positive cultures, and MDR-positive cultures) to explore risk factors that potentially influenced IET and their association with ABR. Chi-square tests were used to evaluate the bivariate association between IET and each potential risk factor. In the multivariate analysis phase, which included all the above-specified variables in all analyses, random intercept logistic regression models were used to identify IET risk factors. Risk was reported as odds ratios (OR). The analyses were stratified by ABR and MDR to examine risk factors that significantly predicted IET for resistant bacteria. All statistical tests were performed using a pre-specified two-tailed alpha level of 0.05. Analyses were conducted using R (R Ver. 4.1.2, R Foundation for Statistical Computing, Vienna, Austria), with RStudio (Boston, MA).

## Results

The majority (65.1%) of the 269 hospitals included in the study database were in urban locations and 66.9% were non-teaching hospitals (Table 1). Overall, 278,344 patients with a positive bacterial culture were evaluated. For most patients, positive cultures were due to gram-negative bacteria (n = 226,725; 81.5%) and Enterobacterales was the most common bacterial pathogen (n = 180,450; 64.8%). Gram-positive bacteria were identified in 71,269 patients (25.6%). There were 19,650 patients (7.1%) with both gram-negative and gram-positive bacteria included in the previous case counts. Amongst patient admissions, 93,252 (33.5%) and 39,000 (14.0%) met the criteria for ABR and MDR, respectively. Gram-negative bacteria accounted for 84,398 (90.5%) ABR admissions and 25,360 (65.0%) MDR admissions.

**Table 1:** Facility demographics

Hospital demographics	N	%
**Total**	**269**	**100%**
Bed size		
< 100	96	35.69%
100–300	108	40.15%
> 300	65	24.16%
Location		
Urban	175	65.06%
Rural	94	34.94%
Teaching status		
Teaching	89	33.09%
Non-teaching	180	66.91%
Census region^z^		
East North Central	42	15.61%
East South Central	36	13.38%
Middle Atlantic	38	14.13%
Mountain	11	4.09%
New England	5	1.86%
Pacific	25	9.29%
South Atlantic	41	15.24%
West North Central	16	5.95%
West South Central	55	20.45%

^a^States included in census regions were East North Central: IL, IN, MI, OH, WI; East South Central: AL, KY, MS, TN; Middle Atlantic: NJ, NY, PA; Mountain: AZ, CO, ID, MT, NM, NV, UT, WY; New England: CT, MA, ME, NH, RI, VT; Pacific: AK, CA, OR, WA; South Atlantic: DE, DC, FL, GA, MD, NC, SC, VA, WV; West North Central: IA, KS, MN, MO, ND, NE, SD; West South Central: AR, LA, OK, TX

### IET by ABR status and clinical characteristics

A total of 56,733 (20.4%) patient admissions with a positive culture received IET. IET occurred in 32,561 (34.9%) and 17,554 (45.0%) of ABR and MDR positive cultures, respectively. Compared with the full cohort, ABR and MDR positive cultures were significantly associated with IET (*P* < 0.001) (Table 2).

**Table 2:** IET in patients with positive bacterial cultures by ABR status. Observed data are presented as n IET admissions/N admissions with positive cultures (%). P values compare the bivariate statistical difference among IET rates across ABR groups (all, ABR, and MDR)

Characteristic	All positive cultures	ABR-positive cultures	MDR-positive cultures	P value across groups
**Overall IET**	56,733/278,344 (20.4%)	32,561/93,252 (34.9%)	17,554/39,000 (45.0%)	< 0.001
**Source**				
Respiratory	6,400/20,598 (31.1%)	4,111/9,217 (44.6%)	2,238/4,439 (50.4%)	< 0.001
Intraabdominal	936/3,021 (31.0%)	444/996 (44.6%)	219/411 (53.3%)	< 0.001
Other	1,258/4,490 (28.0%)	678/1,656 (40.9%)	321/692 (46.4%)	< 0.001
Skin	10,117/39,014 (25.9%)	5,624/13,120 (42.9%)	2,900/6,404 (45.3%)	< 0.001
Multiple	701/2,900 (24.2%)	629/1,723 (36.5%)	334/788 (42.4%)	< 0.001
Urine	31,453/161,874 (19.4%)	17,981/54,502 (33.0%)	9,343/19,588 (47.7%)	< 0.001
Blood	5,868/46,447 (12.6%)	3,094/12,038 (25.7%)	2,199/6,678 (32.9%)	< 0.001
**Onset**				
Hospital	11,679/42,344 (27.6%)	6,716/15,224 (44.1%)	4,353/9,509 (45.8%)	< 0.001
Community	45,054/236,000 (19.1%)	25,845/78,028 (33.1%)	13,201/29,491 (44.8%)	< 0.001
**Pathogen type**				
Both gram negative and gram positive	8,835/19,650 (45.0%)	5,468/11,101 (49.3%)	2,861/5,912 (48.4%)	< 0.001
Gram positive	20,177/71,269 (28.3%)	9,455/19,955 (47.4%)	3,536/7,728 (45.8%)	< 0.001
Gram negative	45,391/226,725 (20.0%)	28,574/84,398 (33.9%)	11,157/25,360 (44.0%)	< 0.001
**Specific pathogen**				
S. *maltophilia* or *Acinetobacter* complex	1,502/2,311 (65.0%)	780/1,124 (69.4%)	573/834 (68.7%)	< 0.001
Multiple bacterial pathogens	12,086/26,904 (44.9%)	7,697/15,470 (49.8%)	4,039/8,126 (49.7%)	< 0.001
*Enterococcus* spp.	8,740/20,821 (42.0%)	2,595/4,346 (59.7%)	2,406/3,956 (60.8%)	< 0.001
*P. aeruginosa*	6,795/18,446 (36.8%)	3,224/6,922 (46.6%)	1,448/2,538 (57.1%)	< 0.001
Enterobacterales	25,415/180,450 (14.1%)	17,151/61,709 (27.8%)	8,164/20,432 (40.0%)	< 0.001
* S.aureus* or *S. pneumoniae*	2,195/29,412 (7.5%)	1,114/3,681 (30.3%)	924/3,114 (29.7%)	< 0.001

Definitions: ABR = antibiotic resistant; IET = inadequate empiric therapy; MDR = multidrug resistant

Evaluations of the association between bacterial pathogen source and incidence of IET found that respiratory or intraabdominal sources had the highest rates of IET. This pattern was consistent across ABR and MDR cultures. Hospital-onset bacterial cultures had a significantly higher rate of IET compared with community-onset cultures overall and for ABR isolates (both P < 0.001), but not for MDR isolates, which had high rates of IET for both community-onset (44.8%) and hospital-onset (45.8%) cultures (P = 0.084).

For all bacterial pathogens, IET occurred at higher rates in the ABR and MDR subgroups compared with the overall patient cohort. IET rates were higher for gram-positive versus gram-negative bacteria, and highest in patients with both gram-positive and gram-negative positive cultures (Table 2). Although *S. maltophilia*/*Acinetobacter* complex accounted for < 1% of positive cultures, the IET rate for this combined bacterial pathogen group was 65.0% in the overall patient cohort. High IET rates were also observed in patients with multiple bacterial bacteria (44.9%), *P. aeruginosa* (36.8%), and *Enterococcus* spp (42.0%). The largest disparities in IET rates based on resistance status were observed for Enterobacterales spp. (14.1% overall, 27.8% for ABR, and 40.0% for MDR) and *S. aureus*/*S. pneumoniae* (7.5% overall, 30.3% for ABR, and 29.7% for MDR).

### IET by SARS-CoV-2 status and time period

**Fig. 1:**
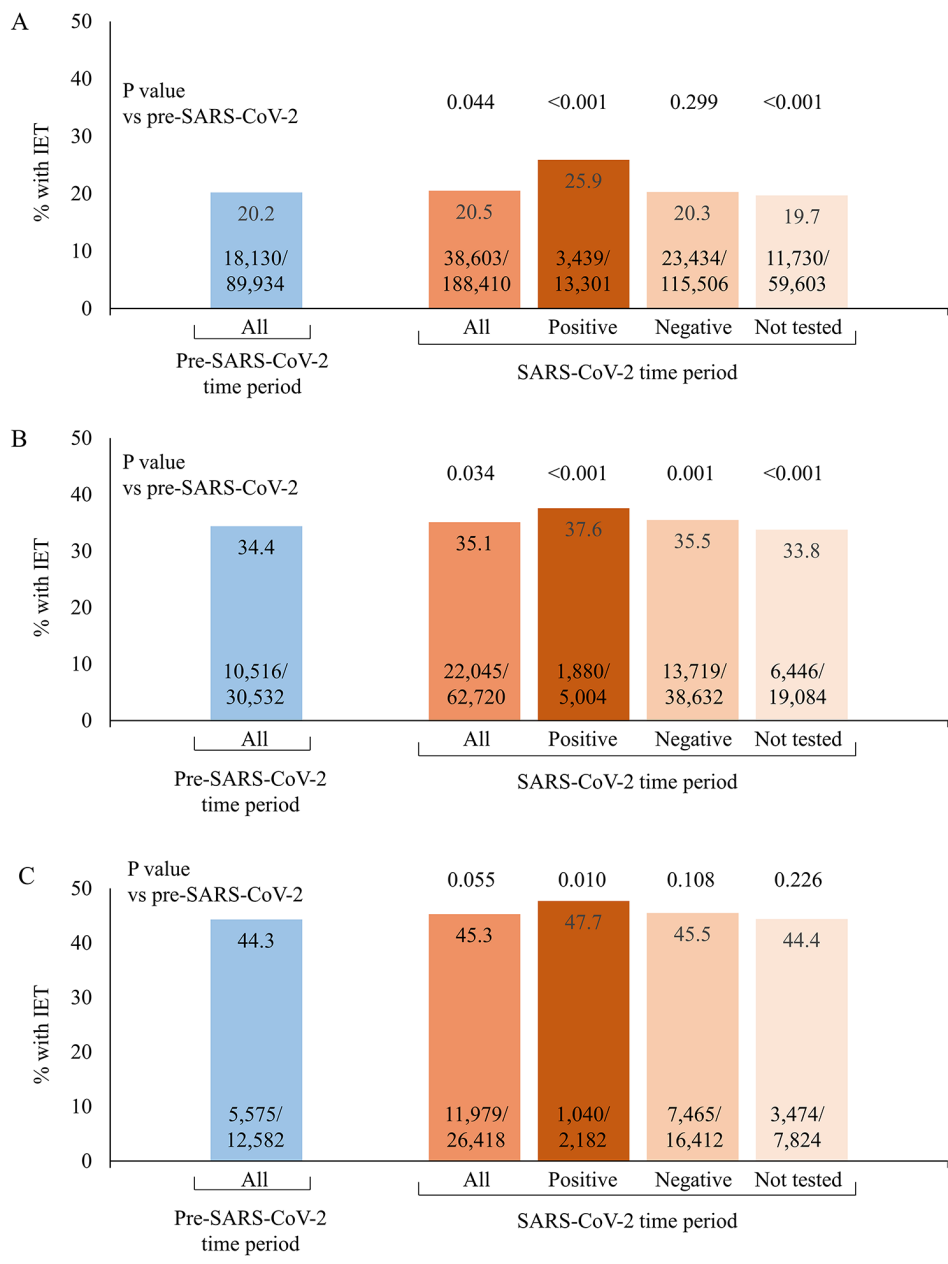
Inadequate empiric therapy (IET) in patients with positive bacterial cultures by SARS-CoV-2 status. for (**A**) All positive cultures; (**B**) Antibiotic-resistant positive cultures; and (**C**) Multi-drug-resistant positive cultures. Observed data are presented as n IET admissions/N admissions with positive cultures (%). “Positive” and “negative” refer to SARS-COV-2-positive and -negative. P values compare the statistical difference in IET rates vs. the pre-SARS-CoV-2 time period

IET rates were slightly, but significantly, higher in the SARS-CoV-2 period (20.5%) compared with the pre-SARS-CoV-2 time period (20.2%; P = 0.044) (Fig. 1). This difference was retained in patients with ABR-positive cultures (35.1% vs. 34.4%; P = 0.034), but did not reach statistical significance in patients with MDR-positive cultures (45.3% vs. 44.3%; P = 0.055). During the SARS-CoV-2 period, significantly higher IET rates compared with the pre-pandemic period were observed in SARS-CoV-2 positive patients, and significantly lower rates were observed in patients not tested for SARS-CoV-2; there was no significant difference in IET rates in SARS-CoV-2-negative patients compared with pre-pandemic rates. For ABR positive cultures, IET rates for all three SARS-CoV-2 testing statuses (positive, negative, and not tested) were significantly higher compared with the pre-pandemic period. For MDR positive cultures, only SARS-CoV-2-positive patients had significantly higher IET rates compared with the pre-pandemic period (Fig. 1).

### Impact of IET on DOT and LOS

Patients who received IET had a significantly longer duration of therapy (mean DOT of 8.86 [standard deviation (SD) 9.03]) compared with patients who received adequate empiric therapy (6.79 [6.29]; P < 0.001). Duration of therapy was longer for IET patients with positive cultures for ABR (9.95 DOT [10.36]) or MDR (10.49 DOT [10.80]; P < 0.001 for both) compared with overall positive cultures (Table 3). More DOT for IET vs. AET was observed for all drug classes for all positive cultures and ABR-positive cultures. In patients with MDR-positive cultures, more DOT with IET vs. AET were observed for FQ, beta-lactamase inhibitor combinations, and macrolides, but not for ESC, carbapenems, and glycopeptides. Patients who received IET, including those with ABR- or MDR-positive cultures, also had significantly increased hospital LOS and ICU LOS compared with those receiving adequate empiric therapy (Table 3).

**Table 3:** Antibacterial duration and hospital/ICU LOS by AET and IET. DOT and LOS data are presented as mean ± standard deviation (interquartile range; median)

Outcome	All positive cultures (N = 278,344 patients)		P value	ABR-positive cultures (N = 93,252 patients)		P value	MDR-positive cultures (N = 39,000 patients)		P value
	AET	IET		AET	IET		AET	IET	
Overall DOT	6.79 ± 6.29 (3–8; 5)	8.86 ± 9.03 (4–11; 6)	< 0.001	8.10 ± 8.04 (4–10; 6)	9.95 ± 10.36 (4–12; 7)	< 0.001	9.89 ± 9.52 (4–12; 7)	10.49 ± 10.80 (4–13; 7)	< 0.001
DOT by antibiotic									
ESC	3.91 ± 3.86 (2–5; 3)	4.33 ± 5.13 (2–5; 3)	< 0.001	4.28 ± 4.81 (2–5; 3)	4.48 ± 5.77 (2–5; 3)	< 0.001	4.52 ± 6.22 (1–6; 3)	4.49 ± 6.12 (2–5; 3)	0.212
Carbapenem	5.04 ± 5.44 (2–6; 3)	5.78 ± 6.77 (2–7; 4)	< 0.001	5.70 ± 5.87 (2–7; 4)	5.88 ± 6.82 (2–7; 4)	< 0.001	6.07 ± 6.17 (3–7; 5)	5.88 ± 7.04 (2–7; 4)	0.423
FQ	3.25 ± 3.75 (1–4; 2)	3.65 ± 4.89 (1–5; 2)	< 0.001	3.72 ± 4.97 (1–5; 2)	3.93 ± 5.41 (1–5; 2)	< 0.001	4.10 ± 5.18 (1–5; 3)	4.29 ± 6.15 (1–5; 3)	0.007
BLI combination	4.06 ± 4.37 (2–5; 3)	5.06 ± 6.19 (2–6; 3)	< 0.001	4.42 ± 5.14 (2–6; 3)	5.21 ± 6.60 (2–7; 3)	< 0.001	4.57 ± 5.56 (2–6; 3)	5.36 ± 6.86 (2–7; 3)	< 0.001
Glyco-peptides	3.19 ± 4.09 (1–4; 2)	4.05 ± 5.01 (1–5; 3)	< 0.001	4.16 ± 5.63 (1–5; 3)	4.48 ± 5.55 (1–6; 3)	0.005	4.98 ± 6.52 (1–6; 3)	4.74 ± 5.79 (1–6; 3)	0.365
Macrolides	2.93 ± 3.61 (1–4; 2)	3.59 ± 4.94 (2–4; 3)	< 0.001	3.27 ± 4.48 (1–4; 3)	3.93 ± 5.74 (1–4; 3)	< 0.001	3.55 ± 5.62 (1–4; 3)	4.18 ± 6.35 (1–5; 3)	< 0.001
LOS days	9.04 ± 10.36 (4–10; 6)	12.24 ± 15.28 (4–14; 7)	< 0.001	10.72 ± 12.99 (4–12; 6)	13.69 ± 17.62 (5–16; 8)	< 0.001	13.06 ± 14.94 (5–15; 8)	14.40 ± 17.83 (5–17; 9)	< 0.001
ICU LOS Days	6.99 ± 8.77 (2–8; 4)	9.39 ± 12.11 (2–12; 5)	< 0.001	8.50 ± 10.95 (2–11; 4)	10.62 ± 13.77 (2–14; 5)	< 0.001	9.95 ± 12.24 (2–13; 5)	11.00 ± 13.58 (3–14; 6)	< 0.001

Definitions: AET = adequate empiric therapy; ABR = antibiotic resistant; DOT = days of therapy; ESC = extended-spectrum cephalosporins; FQ = fluoroquinolones; BLI = Beta-lactamase inhibitor; ICU = intensive care unit; IET = inadequate empiric therapy; LOS = length of stay; MDR = multidrug resistant

### Risk factors for IET in multivariate analyses

A multivariate analysis was performed to determine risk factors associated with IET. Significant increases in the risk of IET were observed for ABR-positive cultures vs. all positive cultures (OR 2.59 [95% CI 2.52–2.65]; P < 0.001) and for MDR-positive cultures vs. all positive cultures (OR 1.84 [95% CI 1.78–1.89]; P < 0.001) (Table 4). MDR-positive cultures were also associated with an increased risk of IET relative to ABR-positive cultures (OR 1.86 [95% CI 1.81–1.92]; P < 0.001). Patients positive for SARS-CoV-2 had a significantly increased risk for IET in the overall cohort (OR 1.20 [95% CI 1.15–1.26]; P < 0.001) and in subgroups with an ABR-positive culture (OR 1.07 [95% CI 1.02–1.12]; P = 0.005) or MDR-positive culture (OR 1.13 [95% CI 1.03–1.25]; P = 0.013).

**Table 4:** Multivariate results for risk factors for IET by ABR status

Factor	All positive cultures		ABR-positive cultures		MDR-positive cultures	
	OR (CI)	P	OR (CI)	P	OR (CI)	P
**ABR**	2.59 (2.52–2.65)	< 0.001	NA	NA	NA	NA
**MDR**	1.84 (1.78–1.89)	< 0.001	1.86 (1.81–1.92)	< 0.001	NA	NA
**SARS-CoV-2 Status**						
Pre SARS-CoV-2	Ref		Ref		Ref	
SARS-CoV-2 positive	1.20 (1.15–1.26)	< 0.001	1.07 (1.02–1.12)	0.005	1.13 (1.03–1.25)	0.013
SARS-CoV-2 negative	0.99 (0.97–1.01)	0.564	1.01 (0.98–1.04)	0.396	1.03 (0.98–1.08)	0.237
SARS-CoV-2 not tested	1.01 (0.98–1.04)	0.651	0.99 (0.96–1.03)	0.621	1.01 (0.95–1.07)	0.814
**Source**						
Other^a^	Ref		Ref		Ref	
Respiratory	1.24 (1.18–1.29)	< 0.001	1.05 (1.00-1.09)	0.042	1.11 (1.02–1.20)	0.021
Urine	0.92 (0.89–0.95)	< 0.001	1.01 (0.96–1.05)	0.767	1.23 (1.16–1.31)	< 0.001
Blood	0.48 (0.46–0.50)	< 0.001	0.66 (0.63–0.70)	< 0.001	0.68 (0.62–0.73)	< 0.001
**Onset**						
Community-onset	Ref		Ref		Ref	
Hospital-onset	1.13 (1.10–1.17)	< 0.001	1.08 (1.03–1.12)	< 0.001	0.89 (0.84–0.94)	< 0.001
**Pathogen type**						
Gram negative only	Ref		Ref		Ref	
Both gram negative and gram positive	1.70 (1.63–1.77)	< 0.001	2.19 (1.88–2.55)	< 0.001	1.98 (1.66–2.36)	< 0.001
Gram positive only	1.62 (1.57–1.66)	< 0.001	0.35 (0.29–0.41)	< 0.001	0.30 (0.25–0.37)	< 0.001
**Specific pathogen**						
*S. aureus* or *S. pneumoniae*	Ref		Ref		Ref	
*Enterococcus* spp.	8.65 (8.18–9.14)	< 0.001	2.92 (2.65–3.22)	< 0.001	2.91 (2.62–3.24)	< 0.001
*S. maltophilia* or *Acinetobacter* complex	5.40 (4.56–6.39)	< 0.001	1.63 (1.31–2.03)	< 0.001	1.33 (1.02–1.72)	0.032
Multiple bacterial pathogens	2.35 (2.06–2.68)	< 0.001	1.11 (0.94–1.30)	0.233	0.93 (0.87–1.02)	0.127
*P. aeruginosa*	1.99 (1.72–2.30)	< 0.001	0.83 (0.69–0.99)	0.048	0.76 (0.60–0.95)	0.018
Enterobacterales	0.58 (0.51–0.68)	< 0.001	0.40 (0.33–0.47)	< 0.001	0.37 (0.30–0.47)	< 0.001
**Underlying condition**						
No underlying condition	Ref		Ref		Ref	
Liver dysfunction	1.11 (1.08–1.13)	< 0.001	1.08 (1.05–1.11)	< 0.001	1.06 (1.01–1.10)	0.021
Heart failure or myocardial injury	1.06 (1.03–1.09)	< 0.001	1.08 (1.04–1.11)	< 0.001	1.11 (1.06–1.17)	< 0.001
Renal failure/ insufficiency	1.06 (1.04–1.09)	< 0.001	1.03 (0.99–1.06)	0.118	1.07 (1.02–1.12)	0.005
Diabetes	1.01 (0.98–1.03)	0.165	1.00 (0.97–1.03)	0.937	1.00 (0.95–1.05)	0.989
Cytokine release syndrome	0.99 (0.96–1.01)	0.900	0.98 (0.94–1.03)	0.432	0.94 (0.88-1.00)	0.065
Sepsis	0.98 (0.94–1.02)	0.263	0.98 (0.96–1.01)	0.648	0.95 (0.90–1.02)	0.165
**Age**						
18–54 years (Q1)	Ref		Ref		Ref	
55–69 years (Q2)	1.06 (1.03–1.09)	< 0.001	1.05 (1.01–1.10)	0.011	1.06 (1.01–1.13)	0.032
70–79 years (Q3)	1.09 (1.06–1.13)	< 0.001	1.06 (1.02–1.11)	0.006	1.10 (1.04–1.17)	0.001
≥ 80 years (Q4)	1.10 (1.06–1.13)	< 0.001	1.01 (0.97–1.06)	0.540	1.13 (1.06–1.20)	< 0.001
**Other characteristics**						
Prior 30-day admission	1.11 (1.07–1.16)	< 0.001	1.17 (1.11–1.22)	< 0.001	1.12 (1.04–1.20)	0.001
Prior 90-day admission	1.10 (1.06–1.13)	< 0.001	1.12 (1.07–1.16)	< 0.001	1.05 (1.00-1.10)	0.050
Female	1.03 (1.01–1.05)	0.013	1.08 (1.05–1.12)	< 0.001	1.11 (1.06–1.16)	< 0.001
Ventilated or ICU	1.02 (0.98–1.06)	0.244	0.97 (0.94–1.01)	0.114	0.97 (0.92–1.02)	0.217
Antibiotic days^b^	1.02 (1.02–1.02)	< 0.001	1.01 (1.01–1.01)	< 0.001	1.01 (1.00-1.01)	< 0.001
LOS days^b^	1.00 (1.00-1.01)	< 0.001	1.01 (1.00-1.01)	< 0.001	1.01 (1.00-1.01)	< 0.001

Definitions: ABR = antibiotic resistant; CI = confidence interval; ICU = intensive care unit; IET = inadequate empiric therapy; LOS = length of stay; MDR = multidrug resistant; OR = odds ratio; Q = quarter; Ref = reference group; SARS-CoV-2 = severe acute respiratory syndrome-coronavirus-2

Data are presented OR (CI). Data for hospital characteristics and geographic location are presented in Supplemental Table S1. Multivariate results were adjusted for all variables shown here and in Supplemental Table S1.

^a^Intra-abdominal, skin/wound, and cultures not defined by the specified categories.

^b^Increase in odds for each additional day.

Additional risk factors for increased IET in patients with a positive bacterial culture included respiratory source, hospital onset, gram-positive or combined gram-positive/gram-negative culture, *Enterococcus* spp., *P. aeruginosa* or *S. maltophilia*/*Acinetobacter* complex, multiple bacterial pathogens, heart failure/myocardial injury, liver dysfunction, renal failure/insufficiency, older age (> 54 years), female sex, more antibiotic DOT, longer hospital LOS, and prior 30-day or 90-day admissions (Table 4). Most of the risk factors for increased IET were retained in subgroup analyses of ABR and MDR (Table 4, Table S1).

## Discussion

Our study spanning 8 months prior to the COVID-19 pandemic through October 2021 and encompassing almost 300,000 hospital admissions with positive bacterial cultures found that SARS-CoV-2-positive patients had significantly higher rates of antibiotic IET compared with the overall patient population during the pre-pandemic period and with SARS-CoV-2-negative and not tested patients during the pandemic period. We further observed elevated IET rates in patients with ABR- or MDR-positive cultures; the IET rate for MDR bacteria in this study (45% for evaluated bacteria across all culture sources) was similar to rates recently reported for patients with bloodstream infections caused by carbapenem-resistant Enterobacterales (44.7%) or VRE (39.6%) [7], providing further confirmation of these data. Our finding that IET was associated with additional DOT and longer hospital and ICU stays is consistent with other studies[1–3, 5, 6, 22] and documents the substantial burden not only for patients receiving IET, but also for hospital facilities, particularly during surge capacity periods.

It is clear from our data that ABR is closely connected with IET. We found a 2.59-fold increased risk of IET with ABR-positive cultures and an MDR-positive culture was associated with an additional 1.86-fold increase in IET compared with ABR-positive cultures. Over one-third (34.9%) of patients with ABR-positive cultures and 45.0% of patients with MDR-positive cultures received IET upon hospital admission, a finding that highlights difficulties in choosing empiric therapy for patients with potentially resistant bacterial pathogens. Of the many factors we assessed in multivariate analyses, including age, comorbidities, isolate source, and ventilator/ICU status, only specific, highly-resistant bacteria (*Enterococcus* and *S. maltophilia*/*Acinetobacter* complex) were associated with a higher risk of IET than ABR. Other factors with significant contributions to high IET rates included respiratory source, hospital-onset infections, and certain underlying conditions (heart failure/myocardial injury, liver dysfunction, and renal failure/insufficiency), older age, and prior admissions. It should be noted that although we identified multiple significant risk factors for IET, the magnitude of their effects varied substantially. IET risk factors identified in our study may serve as predictors of patients in need of aggressive initial antibiotic treatment and as candidates for future studies aimed at optimizing initial therapy choices in high-risk patients.

The SARS-CoV-2 pandemic has further exacerbated challenges with ABR and IET. We previously reported that ABR rates for hospital-onset bacterial infections during the first 20 months of the COVID-19 pandemic were significantly higher than in the preceding pre-pandemic period, particularly in SARS-CoV-2-positive patients, but overall ABR rates were significantly lower in hospitalized patients due to decreased rates in community-onset infections [17]. Although overall IET rates remained similar in the pre-pandemic and pandemic periods, during the SARS-CoV-2 period we observed significant increases in IET over pre-pandemic rates in patients with ABR-positive cultures and those who were SARS-CoV-2 positive; hospital-onset infections were a key contributing factor. Patients with SARS-CoV-2 and bacterial infections are at higher risk for mortality than SARS-CoV-2-negative patients [1, 23] and have more antibiotic usage and longer hospital and ICU LOS [8, 17]. IET, which is associated with increased LOS and mortality [1, 7], likely contributes to these impaired outcomes in SARS-CoV-2-positive patients.

The strong association between ABR and IET was retained during both the pre-pandemic and pandemic periods. A recent report estimated that bacterial ABR was associated with an estimated 4.95 million deaths worldwide in 2019 [24]. There is evidence that the COVID-19 pandemic may have contributed to increases in bacterial ABR in hospitalized patients [17, 25]. During peak capacity periods associated with the COVID-19 pandemic, hospital systems were forced to decrease diagnostic and antibiotic susceptibility testing and reallocate staff from antimicrobial stewardship activities to COVID-related priorities while at the same time increasing antibiotic consumption [13]. The increased ABR rates we have observed in hospitalized SARS-CoV-2-positive patients may reflect these factors, particularly the increased antibiotic exposure and LOS in this patient population [17]. Future longitudinal studies will be needed to explore ongoing changes in IET and ABR in US hospitals. A study of 38 Michigan hospitals found that early increases in antibiotic consumption diminished over time as more experience was gained with managing SARS-CoV-2 infections [26], perhaps augmented by the less severe disease observed later in the pandemic [27]. Lag times of approximately 3 to 6 months have been reported between antibiotic changes and resistance levels in a pre-pandemic study [28], so reductions in ABR may not be immediately apparent.

Irrespective of pandemic-related factors, however, IET remains an important challenge in the treatment of hospitalized patients. Although one option to address this problem is expanded use of broad-spectrum antibiotics, these antibiotics are associated with increased ABR and therefore have the potential to actually compound IET challenges. In addition, broad-spectrum drugs can have negative clinical consequences, including increased risk of *Clostridioides difficile* infection [29, 30] and higher rates of severe sepsis following hospital discharge [31]. Accordingly, the solution to IET does not appear to be indiscriminate broad-spectrum antibiotic use, but rather a tailored therapy approach based on risk factor assessment, diagnostic testing, and antimicrobial stewardship efforts, including vaccination programs to reduce infectious diseases [32–35]. Although the study reported here focused on IET in culture-positive patients, it is important to note that a substantial proportion of culture-negative or not tested hospitalized patients receive prolonged antibiotic therapy (> 3 days) [17]. This patient population would benefit from antimicrobial stewardship programs as well.

Study limitations include the use of facility reports rather than a central laboratory for SARS-CoV-2 and antibiotic susceptibility tests. Different laboratories may have used different testing systems and breakpoints for determination of resistance, thereby potentially affecting the ABR and MDR rates reported here. Analyses were based on positive SARS-CoV-2 tests and not on symptomatic infection, so asymptomatic patients admitted for other causes may have been included. Similarly, patients with positive bacterial cultures did not necessarily have a confirmed bacterial infection. Our testing algorithm was designed to remove admissions with contaminating bacteria [20], but it is possible that some bacteria included in our analyses were colonizers. Due to database limitations, information on outpatient antibiotic exposure was not available and antibiotics prescribed in prior admissions were not evaluated, although we did evaluate whether patients were admitted in the prior 30 and 90 days. Selection bias (e.g., a greater likelihood of collecting bacterial culture data on more severely ill patients) may have influenced reported resistance rates. Certain geographic areas and smaller hospitals may have been underrepresented in our database.

## Conclusion

Our study documents elevated rates of IET in patients with ABR- or MDR-positive cultures and in patients positive for SARS-CoV-2. IET rates appear to be inextricably linked to ABR, and improvements in both are likely to require expanded use of rapid diagnostic tests, intensified vaccination programs, and a renewed commitment to antimicrobial stewardship programs. The data from our study may be of use in focusing future research efforts aimed at improving the adequacy of empiric therapy and reducing ABR in hospitalized patients.

## Electronic supplementary material

Below is the link to the electronic supplementary material.

Supplementary Material 1: Section S1: Antibiotic susceptibility analyses. Table S1: Hospital and geographic multivariate results for risk factors for inadequate therapy by ABR status. Data are presented as odds ratio (OR) (confidence interval [CI]).

## Data Availability

The datasets used and/or analyzed during the current study are available from the corresponding author on reasonable request.
