# Supplementary material for: Association of SARS-CoV-2 status and antibiotic-resistant bacteria with inadequate empiric therapy in hospitalized patients: a US multicenter cohort evaluation (July 2019 - October 2021)
Source: BMC Infect Dis. 2023 Jul 24;23:490. doi: 10.1186/s12879-023-08453-z (PMC10367264; doi:10.1186/s12879-023-08453-z)
Supplement: Supplementary file 1 — Supplementary Material 1: Section S1: Antibiotic susceptibility analyses. Table S1: Hospital and geographic multivariate results for risk factors for inadequate therapy by ABR status. Data are presented as odds ratio (OR) (confidence interval [CI]). [file 12879_2023_8453_MOESM1_ESM.docx]

**Supplemental Information**

**Association of SARS-CoV-2 status and antibiotic-resistant bacteria with
inadequate empiric therapy in hospitalized patients: a US multicenter cohort evaluation
(July 2019 - October 2021)**

Karri A. Bauer^1^, Laura A. Puzniak^1^, Kalvin C. Yu^2^, Kenneth P. Klinker^1^, Janet A. Watts^2^, Pamela A. Moise^1^, Lyn Finelli^1^, and Vikas Gupta^2*^

^1^Merck & Co., Inc., Kenilworth, New Jersey, USA

^2^Becton, Dickinson and Company, Franklin Lakes, NJ, USA

**Supplemental Section S1**. Antibiotic susceptibility analyses.

Antibiotic nonsusceptibility (NS) was defined as a laboratory result of intermediate (I) or resistant (R) and evaluated in gram-negative bacteria for the following antibiotics:

- Extended-spectrum cephalosporins: NS to cefepime or ceftazidime, cefotaxime (excluded for *Pseudomonas aeruginosa* [PsA]/*Acinetobacter* complex [ACB]), or ceftriaxone (excluded for PsA/ACB])
- Piperacillin-tazobactam NS
- Carbapenems: Enterobacterales NS to ertapenem, meropenem, doripenem, or imipenem (excluded for *Proteus mirabilis* and *Morganella morganii*); PsA and ACB NS to imipenem, meropenem, or doripenem; all *Stenotrophomonas maltophilia* were presumed to be NS
- Fluroquinolones: NS to ciprofloxacin, levofloxacin or moxifloxacin (excluded for PsA/ACB)
- Multi-drug resistant: Carbapenem NS, extended-spectrum beta-lactamase producing phenotype, pan-beta-lactam NS, or NS to at least 1 drug in 3 of the following 5 classes: extended-spectrum cephalosporins, fluoroquinolones, aminoglycosides; carbapenems; piperacillin or piperacillin-tazobactam (with exclusions for PsA/ACB as noted above)

Antibiotic susceptibility was evaluated in gram-positive bacteria as follows:

- - *Enterococcus* spp. resistant to vancomycin (VRE)
  - *Staphylococcus aureus* resistant to methicillin (MRSA)
  - *Streptococcus pneumoniae* NS to:
    - Penicillin
    - Macrolides: NS to erythromycin, azithromycin, or clarithromycin
    - Fluoroquinolones: NS to levofloxacin or moxifloxacin
    - Extended-spectrum cephalosporins: NS to ceftriaxone, cefotaxime, or cefepime
    - Tetracyclines: NS to doxycycline or tetracycline
  - Multi-drug resistant: VRE or MRSA

**Supplemental Table S1.** Hospital and geographic multivariate results for risk factors for inadequate therapy by ABR status. Data are presented as odds ratio (OR) (confidence interval [CI]).

| **Factor** | **All positive cultures IET** | | **ABR-positive cultures IET** | | **MDR-positive cultures IET** | |
| --- | --- | --- | --- | --- | --- | --- |
|  | **OR (CI)** | **P** | **OR (CI)** | **P** | **OR (CI)** | **P** |
| **Hospital-level characteristics** |  |  |  |  |  |  |
| Urban (vs rural) | 1.02  (0.95-1.09) | 0.733 | 1.02  (0.98-1.07) | 0.653 | 1.08  (0.98-1.20) | 0.123 |
| Teaching (vs non-teaching) | 1.03  (0.99-1.07) | 0.130 | 1.04  (0.98-1.09) | 0.483 | 1.04  (0.94-1.15) | 0.427 |
| **Bed size** |  |  |  |  |  |  |
| <100 | Ref |  | Ref |  | Ref |  |
| 100-300 | 0.97  (0.92-1.02) | 0.495 | 0.93  (0.83-1.04) | 0.180 | 0.96  (0.85-1.09) | 0.513 |
| > 300 | 0.89  (0.84-0.95) | 0.003 | 0.87  (0.77-0.98) | 0.035 | 0.96  (0.84-1.10) | 0.566 |
| **Census region** |  |  |  |  |  |  |
| East North Central | Ref |  | Ref |  | Ref |  |
| East South Central | 1.08  (0.98-1.19) | 0.086 | 1.06  (0.91-1.23) | 0.458 | 0.95  (0.81-1.12) | 0.550 |
| Middle Atlantic | 0.92  (0.79-1.07) | 0.271 | 0.87  (0.79-0.95) | 0.008 | 0.84  (0.72-0.98) | 0.027 |
| Mountain | 1.01  (0.91-1.17) | 0.735 | 1.05  (0.97-1.13) | 0.683 | 1.07  (0.93-1.28) | 0.611 |
| New England | 1.11  (0.99-1.30) | 0.060 | 1.12  (1.01-1.34) | 0.048 | 1.18  (1.05-1.31) | 0.028 |
| Pacific | 0.86  (0.76-0.98) | 0.013 | 0.87  (0.74-1.04) | 0.119 | 0.83  (0.70-0.98) | 0.033 |
| South Atlantic | 0.95  (0.85-1.06) | 0.458 | 0.96  (0.93-1.01) | 0.171 | 0.94  (0.81-1.09) | 0.425 |
| West North Central | 0.79  (0.68-0.95) | 0.009 | 0.79  (0.66-0.97) | 0.003 | 0.54  (0.41-0.72) | <0.001 |
| West South Central | 0.89  (0.78-1.02) | 0.123 | 0.92  (0.87-0.97) | 0.027 | 0.87  (0.75-0.99) | 0.048 |

ABR, antibiotic resistant; CI, confidence interval; IET, inadequate empiric therapy; MDR, multidrug resistant; OR, odds ratio; Ref, reference group.
